# Inflammation-Related Genes Serve as Prognostic Biomarkers and Involve in Immunosuppressive Microenvironment to Promote Gastric Cancer Progression

**DOI:** 10.3389/fmed.2022.801647

**Published:** 2022-03-16

**Authors:** Yuanfeng Wei, Limin Gao, Xi Yang, Xiaoyu Xiang, Cheng Yi

**Affiliations:** ^1^Department of Medical Oncology, Cancer Center, West China Hospital, Sichuan University, Chengdu, China; ^2^Department of Pathology, West China Hospital, Sichuan University, Chengdu, China

**Keywords:** tumor microenvironment, immunosuppressive, gastric cancer, prognostic biomarkers, inflammation

## Abstract

Gastric cancer (GC) is a typical inflammatory-related malignant tumor which is closely related to helicobacter pylori infection. Tumor inflammatory microenvironment plays a crucial role in tumor progression and affect the clinical benefit from immunotherapy. In recent years, immunotherapy for gastric cancer has achieved promising outcomes, but not all patients can benefit from immunotherapy due to tumor heterogeneity. In our study, we identified 29 differentially expressed and prognostic inflammation-related genes in GC and normal samples. Based on those genes, we constructed a prognostic model using a least absolute shrinkage and selection operator (LASSO) algorithm, which categorized patients with GC into two groups. The high-risk group have the characteristics of “cold tumor” and have a poorer prognosis. In contrast, low-risk group was “hot tumor” and had better prognosis. Targeting inflammatory-related genes and remodeling tumor microenvironment to turn “cold tumor” into “hot tumor” may be a promising solution to improve the efficacy of immunotherapy for patients with GC.

## Introduction

Global cancer statistics 2020 showed that there were 1,089,103 new cases of gastric cancer (GC) and 768,793 deaths, leading to the 5th and 4th incidence and mortality rates, respectively (1). Recently, although some progress has been made in surgery, chemotherapy, radiotherapy, targeted therapy, anti-angiogenic therapy, and immunotherapy (2), the survival of patients with advanced GC remains dismal (3). Therefore, it is necessary to actively explore its pathogenesis, effective prognostic markers, and elements of poor therapeutic outcome.

Nowadays, although the pathogenesis of GC has not been fully elucidated, various studies have shown that helicobacter pylori (HP) infection is considered to be an essential contributor to the development of GC (4, 5). HP infection could stimulate an inflammatory response in the body, which in turn induces oncogenic mutations and malignant cell transformation, ultimately leading to intestinal epithelial metaplasia, cellular dysplasia, and gastric carcinogenesis (6, 7). In addition, approximately 10% of patients with GC manifest an association with Epstein-Barr virus (EBV) infection (8, 9). This virus is involved in the malignant cells in 80% of patients with lymphoid stromal GC and can also promote inflammatory changes in the gastric mucosa, thereby promoting the development of GC (10, 11). It is evident that chronic inflammation plays a significant part in the progression of GC (12). It could facilitate tumorigenesis and progression through the release of inflammatory mediators that suppresses the immune response, promote mitogenesis, and chemotaxis of cancer cells (13, 14). Inhibition of the associated inflammatory response may be a crucial part of the strategy to tackle GC. However, it is well known that GC subtypes are molecularly distinct, different subtypes of patients with GC have the different characteristics and therapeutic strategies (15, 16). This indicates that the specific inflammatory status of GC subgroups will help the patients to find the suitable treatment. In recent years, there are many new molecular classification methods for GC classification (17, 18). However, the association of inflammation-related genes and prognosis of GC is remained unclear.

Simultaneously, the inflammatory response was closely correlated with the immune status (19). The persistence of inflammation of the tumor in most cases can lead to a depression of the body’s innate immunity, thereby reinforcing tumor-mediated immunity and leading to tumor development, local infiltration, vascular regeneration, and distant metastasis (19–21). Previous studies have also suggested that the potent inflammatory effect is connected to the regulation of the tumor immune microenvironment (22). Thus, understanding the relationship between inflammation-related genes and the prognosis of GC and the interaction of tumor microenvironment (TME) and its relevance to inflammatory response is an attractive area to explore.

Herein, we constructed a prognostic model based on inflammation-related genes of patients with GC. Besides, we analyzed the associations between inflammation-related genes and the tumor immune microenvironment to explore the potential strategies to overcome immunosuppressive tumor microenvironment, and improving the efficiency of immunotherapy. The analysis showed that the prognostic inflammation-related genes may be a potential immunotherapy target and prognostic biomarker for GC.

## Materials and Methods

### Data Extraction

In this study, 375 GC and 32 normal samples were obtained from The Cancer Genome Atlas (TCGA) (discovery cohort) (23). In addition, a total of 433 GC patients were obtained from GSE84437 (validation cohort) (24). The clinical data, such as sex, age, survival time, overall survival (OS) status, and clinical T, N, M stage, were obtained. Next, we obtained the gene sets related to the inflammatory status from the National Center for Biotechnology Information’s (NCBI) gene database^1^, with the keywords “inflammatory and “homo sapiens” [porgn: _txid9606]”(25, 26). Moreover, 200 inflammation-related genes were acquired from the gene set enrichment analysis (GSEA) website (27). Finally, a total of 2,685 inflammation-related genes were obtained from the two datasets for further analyses (Supplementary Table 1).

### Data Preprocessing

Applied Perl and R language, the tumor samples downloaded from the TCGA database and sorted to obtain the expression matrix, followed by the process of converting the gene IDs into gene symbols. Similarly, the expression matrix downloaded from the Gene Expression Omnibus (GEO) database. Finally, the gene expression for each of the above patients were normalized and log2 transformed by the R package “limma.” Specifically, we used the meaning function to process the expression data of all genes and removed genes with zero expression in all samples and the meaning expression value were standardized by log2 transformation. After cleaning and calibrating the data, we adopted the Wilcox test to analyze the differential expressed genes between normal and tumor groups with the criteria of false discovery rate (FDR) < 0.05 and | log FC| > 2 in the discovery cohort and visualized by the R package “ggplot2” and “pheatmap.” After that, the 3,165 differentially expressed genes (DEGs) intersected with 2,685 inflammation-related genes and obtained 215 differentially expressed inflammation-related genes by the R package “venn”.

### Differentially Expressed and Prognostic Inflammation-Related Genes

To investigate the relationship between inflammation-related genes and the OS of patients with GC, we performed the univariate Cox analysis about the 2,685 inflammation-related genes with the threshold of *p* less than 0.05 by the R package “survival.” Subsequently, 354 prognostic inflammation-related genes intersected with the 215 differentially expressed inflammation-related genes by the R package “venn,” we obtained 29 differentially expressed and prognostic inflammation-related genes and visualized by the R package “pheatmap.”

### Construction and Validation of a Prognostic Inflammation-Related Genes Signature

To investigate the relationship between inflammation-related genes and prognosis for GC, we constructed a prognostic model using the least absolute shrinkage and selection operator (LASSO) algorithm analysis by the “glmnet” of R package based on 29 differentially expressed and prognostic inflammation-related genes. Subsequently, we obtained the corresponding coefficients of the 29 genes and the patients’ risk scores were obtained based on the formula: score = e sum(eachgenecorrespondingcoefficient)′sexpression×. According to the formula, if the corresponding coefficients of the genes were zero, there was little significance. Next, we reduced the number of genes with the corresponding coefficient was equal to zero. Among these 29 genes, there were 18 genes with coefficients of zero, so we retained 11 genes for subsequent analysis and some of them were validated by immunochemistry in the Human Protein Atlas (HPA) database^2^. Finally, the patients with GC were divided into two groups based on the median risk score. The principal component analysis (PCA) and t-distributed stochastic neighbor embedding (t-SNE) analysis were conducted by using the “Rtsne” and “ggplot2” R packages to investigate the distribution of different groups. The “survminer” and “survival” R package were used to the survival analysis. A time-dependent receiver operating characteristic (ROC) curve analysis was conducted by the “survival” and “timeROC” R package.

### Functional Enrichment and Immune-Related Analysis Among Two Risk Groups

To further investigate the differences in gene function and pathways between two subgroups, we downloaded the GSEA software (version 4.2.2) from the website^3^ and performed GSEA (27, 28) analysis with the criterion of *p* < 0.05 and FDR < 0.25 (29). The gene set permutations in each analysis were set as 1,000 times and the top five items in each group were presented in the result. The infiltration of immune cells and immune-related pathways were measured by ssGSEA using the R package “GSVA” and “GSEABase.”

### Evaluation of Cell Type Components of the Tumor Microenvironment

CIBERSORT was employed to estimate cell subtypes in the TME of high- and low-risk groups. One sample with the sum of all immune cell types scored equal to 1. CIBERSORT algorithm was applied to analyze the gene expression data by executing 1,000 permutations. The value of *p* < 0.05 was considered statistically significant.

### The Real-Time Quantitative PCR Analysis

A total of 10 cases of formalin-fixed paraffin-embedded GC tissue and paired normal tissues were collected from the Department of Pathology, West China Hospital, Sichuan University from 2020 to 2021 to validate the hub genes (*PON1, MATN3*, and *SERPINE1*). The Ethical Committee of West China Hospital approved this study and waived informed consent. The primers were purchased from the Wuhan Servicebio Technology Co., Ltd., and the experiments were done by them. The forward primer sequences were labeled as “F,” the reverse primer sequences were marked as “R,” and the sequences ordered from 5′ to 3′. The details of primers were as follows: PON1-F: CACCAGTCTTCTTACCAAACACGA, PON1-R: TCTCCAAGTCTTCAGAGCCA GTT, MATN3-F: GAGCCCTCTTCTAACATCCCTAAG, MATN3-R: GGTGT GTT CCAAGCACACAGG, SERPINE1-F:CCCCACTTCTTC AGGCTGTT, SERPINE1-R: GCCGTTGAAGTAGAGGGCAT, GAPDH-F: GGAAGCTTGTCATCAATGGA AATC, and GAPDH-R: TGATGACCCTTTTGGCTCCC. The experiments were performed according to the manufacturer’s instructions. Briefly, the total RNA of each sample was extracted. Subsequently, the RNA was reverse transcribed into cDNA using a Servicebio^®^RT First Strand cDNA Synthesis Kit (Servicebio, Wuhan, China). Then, real-time quantitative PCR (qRT-PCR) reaction was performed on a Real-Time PCR System (Bio-Rad). The relative mRNA expression level was calculated by the 2^–△△CT^ method. The value of *p* < 0.05 was considered statistically significant.

## Results

### Identification of Differentially Expressed and Prognostic Inflammation-Related Genes

**FIGURE 1:**
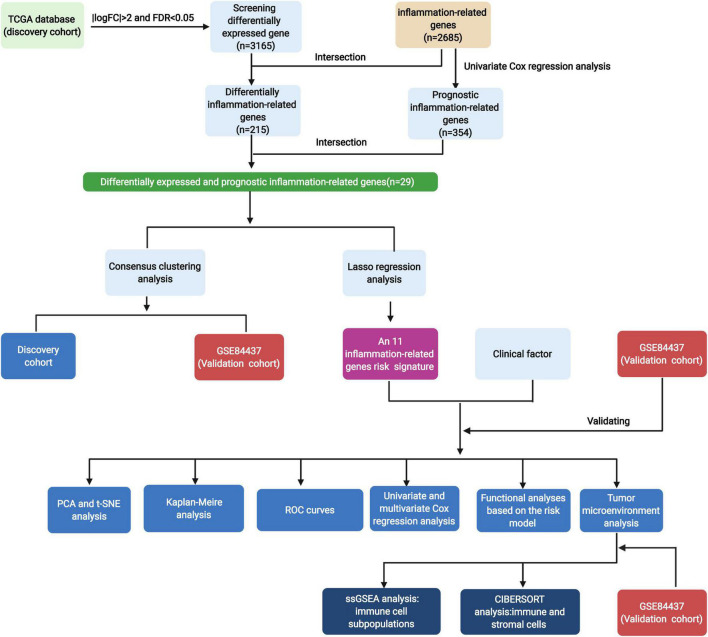
Flowchart of the main procedures of this study.

**FIGURE 2:**
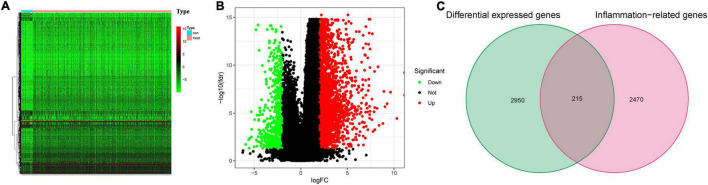
Differentially expressed inflammation-related genes. **(A)** The heatmap for differential expressed genes between normal and patients with gastric cancer (GC). **(B)** Volcano plot. **(C)** Venn diagram to identify differentially expressed inflammation-related genes.

**FIGURE 3:**
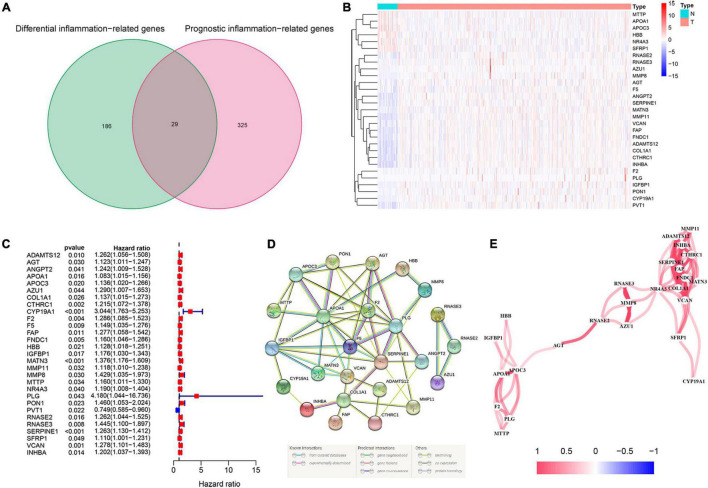
Identification of the candidate inflammation-related genes in the Cancer Genome Atlas (TCGA) cohort. **(A)** Venn diagram to identify differentially expressed and prognostic inflammation-related genes. **(B)** The heatmap of differentially expressed and prognostic inflammation-related genes. **(C)** Forest plots of the association between 29 differentially expressed and prognostic inflammation-related genes and overall survival (OS). **(D)** The protein–protein interaction (PPI) network of differentially expressed and prognostic inflammation-related genes (interaction score = 0.4). **(E)** The correlation network of candidate genes.

The flowchart diagram of the major procedures is shown in Figure 1. First, we obtained 3,165 DEGs between normal and patients with GC (Figures 2A,B). Second, a total of 215 differentially expressed inflammation-related genes were identified by intersecting with the 3,165 DEGs and 2,685 inflammation-related genes (Figure 2C). Third, a univariate Cox analysis showed that 354 inflammation-related genes were related to OS with *p* < 0.05 in a TCGA cohort. Finally, 29 differentially expressed and prognostic inflammation-related genes were obtained by taking the intersection of 354 prognostic inflammation-related genes and 215 differentially expressed inflammation-related genes and visualized using a heatmap (Figures 3A,B). A univariate Cox regression analysis revealed that the genes correlated with OS in patients with GC, including 28 risk genes (Hazard ratio > 1) and one protective gene (HR < 1) (Figure 3C). To further investigate these inflammation-related genes interactions, protein–protein interaction (PPI) analysis was carried out and the result was presented in Figure 3D. We identified *APOA1, APOC3, IGFBP1, MATN3, SERPINE1, F2, F5, and PLG* as hub genes and the correlation among these genes are displayed in Figure 3E.

### Construction of a Prognostic Model in the Discovery Cohort

**FIGURE 4:**
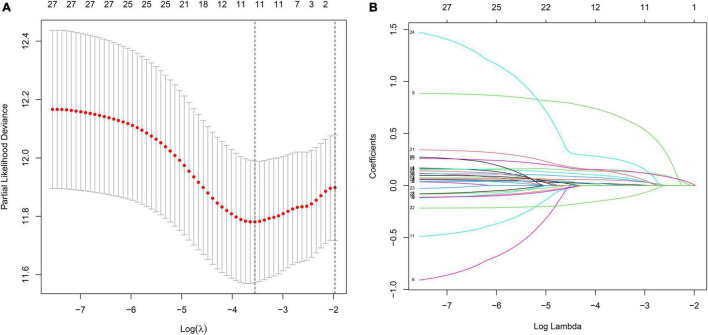
The construction of inflammation-related gene signature for the prediction of prognosis in GC. **(A)** Least absolute shrinkage and selection operator (LASSO) regression. **(B)** LASSO coefficient profiles of 11 inflammation-related genes.

**FIGURE 5:**
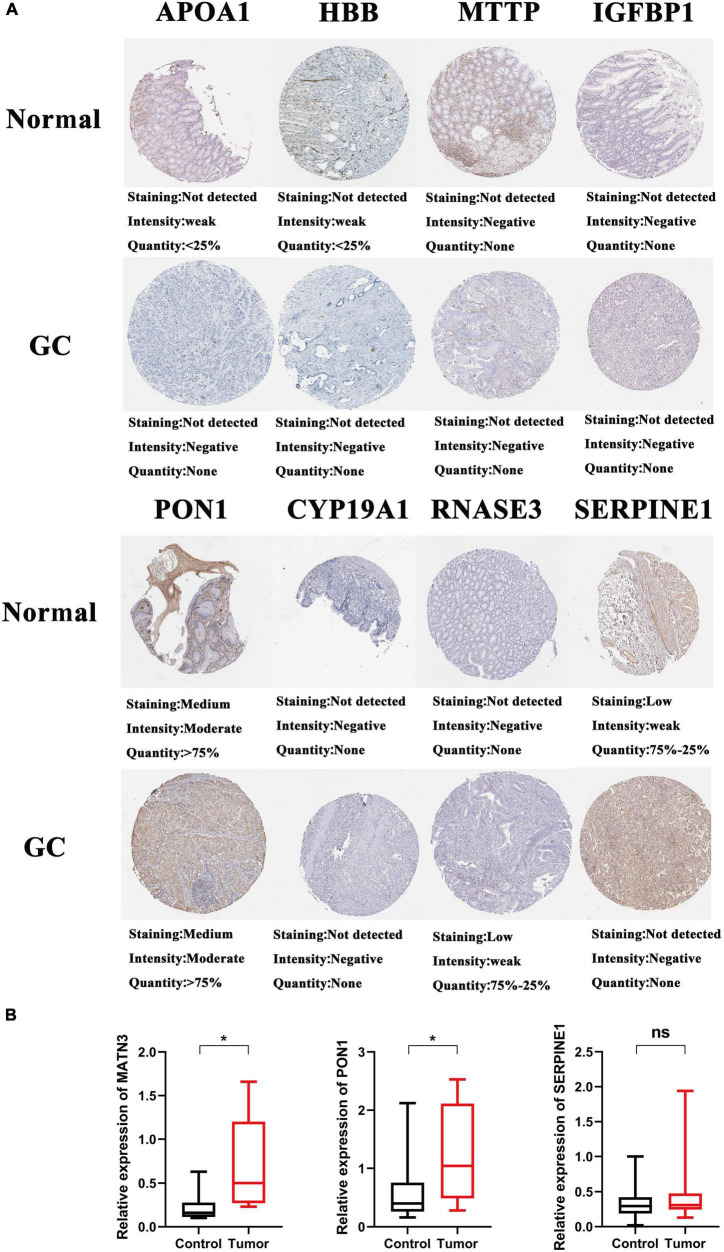
The expression of candidate genes in GC and normal tissue. **(A)** The protein expression of the hub genes in the Human Protein Atlas (HPA) database. **(B)** the mRNA expression of PON1, MATN3, and SERPINE1 in GC and adjacent tissues, *p < 0.05.

**FIGURE 6:**
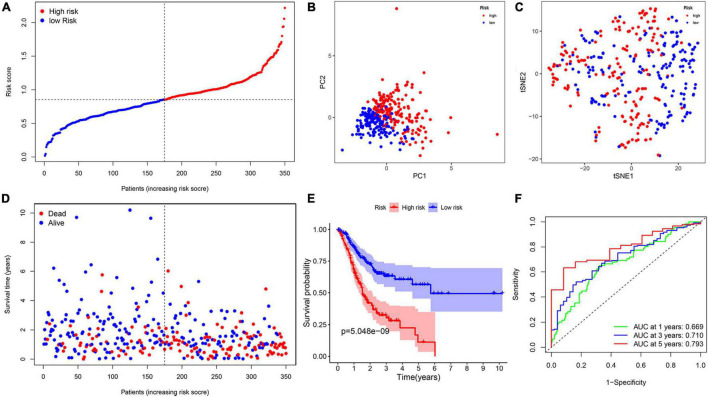
The construction of risk signature in the TCGA cohort. **(A)** The distribution of risk score. **(B)** Principal component analysis (PCA) analysis. **(C)** T-distributed stochastic neighbor embedding (T-SNE) plot. **(D)** The distribution of OS. **(E)** The Kaplan–Meier survival analysis of OS between two groups. **(F)** Area under the curve (AUC) in receiver operating characteristic (ROC) analysis for risk signature at 1-, 3-, and 5-year survival time.

To investigate the relationship between inflammation-related genes and prognosis for patients with GC, a LASSO algorithm analysis was used to construct the prognostic model. In TCGA cohort, the LASSO Cox regression analysis results (Figures 4A,B) showed that a 11-gene signature (*APOA1, CYP19A1, F5, HBB, IGFBP1, MATN3, MTTP, PON1, PVT1, RNASE3, and SERPINE1*) was constructed. Next, we analyzed the expression of them in GC and normal samples in HPA database and the result was presented in Figure 5A. To better reveal the reality of the results of bioinformatic, we have collected GC tissues and non-cancerous tissues to validate the main DEGs by qRT-PCR. The results showed the expression level of *PON1, MATN3, and SERPINE1* were increased in the tumor tissues (Figure 5B). Moreover, there is no significant difference in the expression of *SERPINE1* between GC tissues and normal controls, a larger sample size needs to be implemented to validate our results in future. Depending on the median score, patients were divided into high- and low-risk groups (Figure 6A). The PCA and t-SNE analysis indicated that the patients with GC of high- and low-risk groups had a distinctly two different directions (Figures 6B,C). Moreover, the scatter plot showed that most high-risk patients died relatively earlier compared with low-risk patients (Figure 6D). Consistently, the K–M curve showed patients with high-risk GC had a poor OS (Figure 6E). The sensitivity and specificity of the prognostic model were assessed by applying the time dependent ROC analysis, and we observed that the area under the ROC curve (AUC) at 1, 3, and 5 years were 0.669, 0.710, and 0.793, respectively (Figure 6F).

### External Validation of the Risk Signature

**FIGURE 7:**
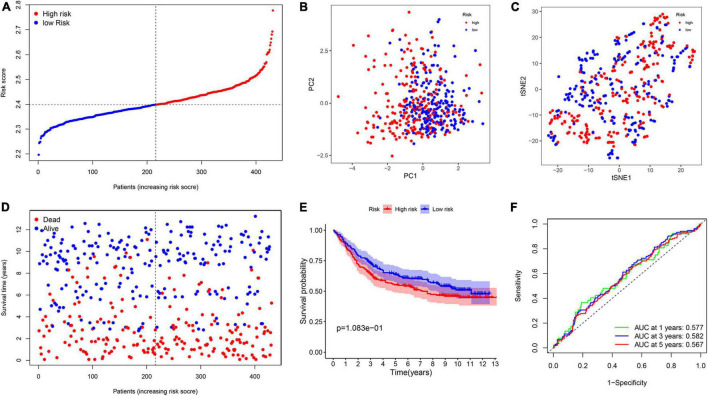
Validation of the prognostic model in the Gene Expression Omnibus (GEO) cohort. **(A)** The distribution of risk score. **(B)** PCA analysis. **(C)** T-SNE plot. **(D)** The distribution of OS. **(E)** The Kaplan–Meier survival analysis between the two groups. **(F)** AUC in ROC analysis for risk signature at 1-, 3-, and 5-year survival time.

To measure the reliability of the model constructed in the TCGA cohort, a total of 433 patients with GC acquired from GSE84437 cohort were utilized as the validation set. In addition, patients were separated into two groups based on the median value from the TCGA cohort (Figure 7A). The PCA and t-SNE analyses divided patients with GC into two subgroups, which were consistent with results obtained from the TCGA cohort (Figures 7B,C). Patients in the high-risk group were had a worse survival time (Figures 7D,E). Moreover, the AUC at 1, 3, and 5 years were 0.577, 0.582, and 0.567, respectively (Figure 7F).

### Independent Prognostic Value of the Risk Model

**FIGURE 8:**
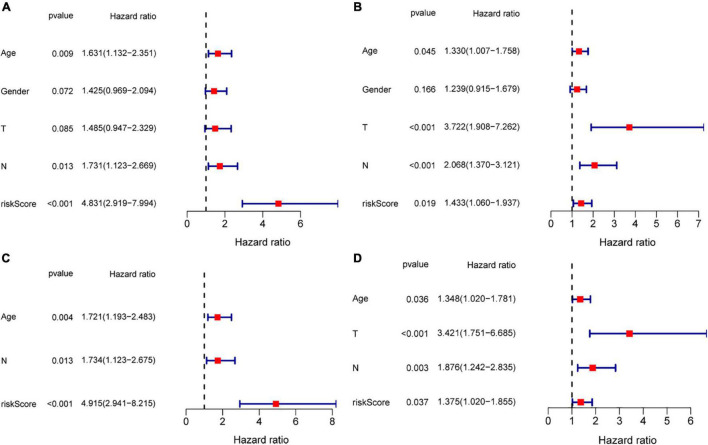
Risk score as an independent prognostic signature for patients with GC. Results of the univariate and multivariate Cox regression analyses of OS in the TCGA cohort **(A,C)** and in the GEO cohort **(B,D)**.

To evaluate the possibility of risk score serving as an independent prognostic indicator. The univariate cox regression analysis demonstrated that in both TCGA and GEO cohorts, the risk score was proved to be an independently significant predictor of poor survival (TCGA: *HR* = 4.831, 95% *CI*: 2.919–7.994, *p* < 0.001; GEO: *HR* = 1.433, 95% *CI*: 1.060–1.937, *p* = 0.019, Figures 8A,B). Consistently, the risk score still indicated to be an independent predictor for OS in the multivariate Cox regression analysis (TCGA: *HR* = 4.915, 95% *CI*: 2.941–8.215, *p* < 0.001; GEO: *HR* = 1.375, 95% *CI*: 1.020–1.855, *p* = 0.037, Figures 8C,D).

### Functional Analyses Based on the Risk Model

**FIGURE 9:**
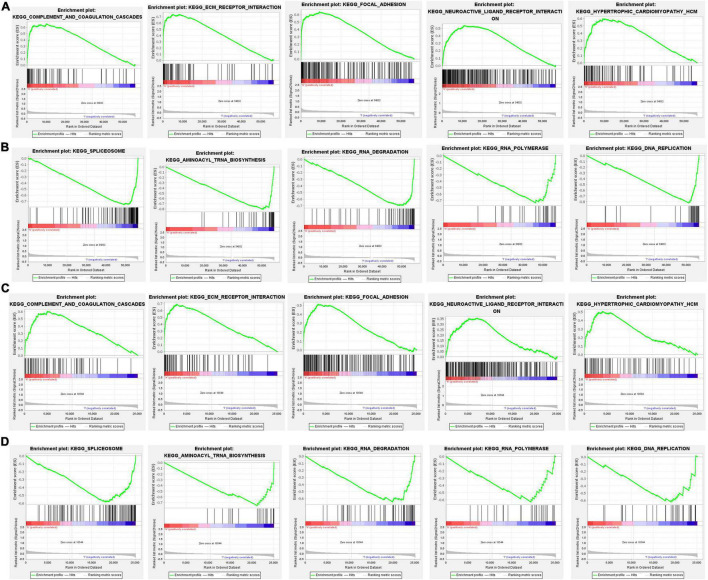
The gene set enrichment analysis (GSEA) analysis between different risk groups. **(A,B)** KEGG enrichment in the TCGA cohort, **(C,D)** Kyoto Encyclopedia of Genes and Genomes (KEGG) enrichment in the GEO cohort.

To further investigate the differences in gene function and pathways between two subgroups, a GSEA analysis was performed. In the TCGA cohort, the results indicated that complement and coagulation cascades, ECM receptor interaction, focal adhesion, neuroactive ligand receptor interaction, and hypertrophic cardiomyopathy HCM were mainly enriched in the high-risk group (Figure 9A). In contrast, spliceosome, aminoacyl-tRNA biosynthesis, RNA degradation, RNA polymerase, and DNA replication were mainly enriched in the low-risk group (Figure 9B). In the GEO cohort, the results were consistent with the result in TCGA cohort (Figures 9C,D).

### Evaluation of the Components of Tumor Microenvironment

**FIGURE 10:**
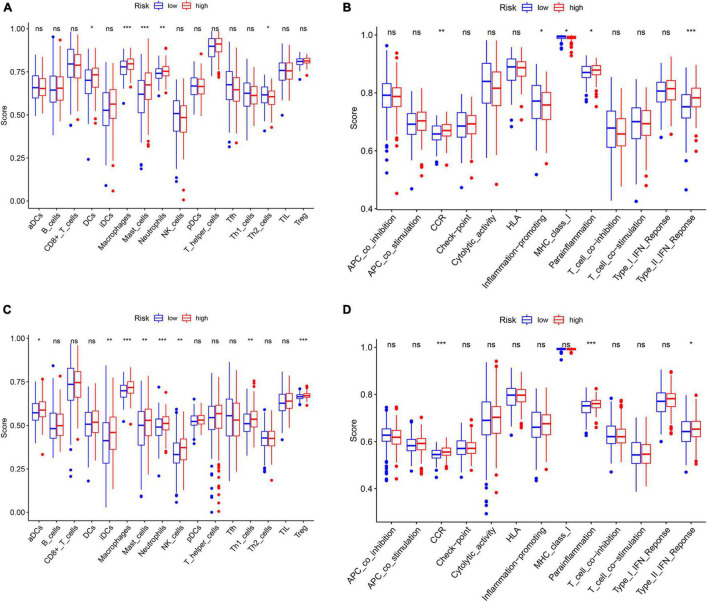
Differences of immune cells infiltration and immune-related pathways between risk groups. **(A)** Immune cells and **(B)** immune-related functions in the TCGA cohort. **(C)** Immune cells **(D)** and immune-related functions in the GEO cohort. ns: not significant; *p < 0.05; **p < 0.01; ***p < 0.001.

**FIGURE 11:**
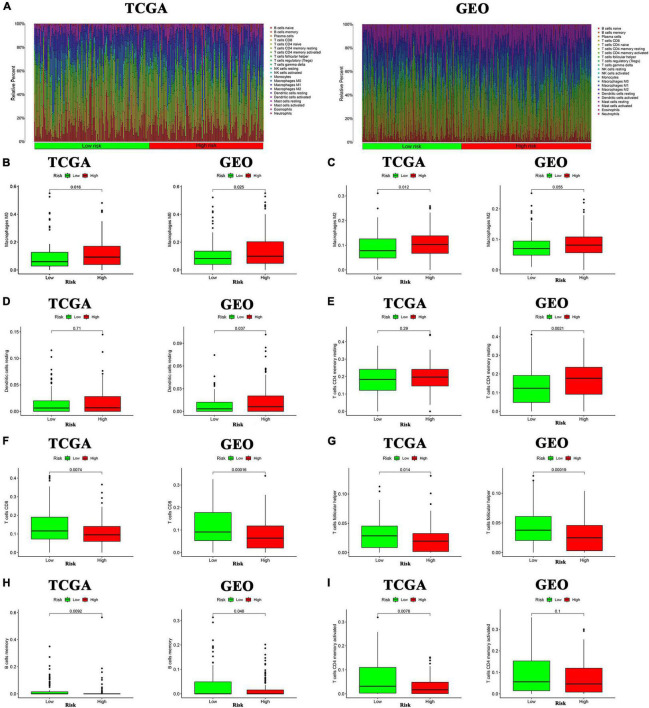
Analysis of immune cells in the high- and low-inflammatory risk group for GC. **(A)** Relative proportion of immune infiltration in high- and low-inflammatory risk patients. **(B–I)** Box plot visualizing the differentially infiltrated immune cells in the two groups.

The ssGSEA was applied to explore the correlation between risk score and the different immune cell subpopulations. In the TCGA cohort, the immune cell subpopulations of dendritic cells (DCs), macrophages, mast cells, and the immune-related pathways, such as chemokine receptors (CCR), major histocompatibility complex (MHC) class I, para-inflammation, and type II interferon (IFN) response were upregulated in the high-risk groups (Figures 10A,B). In the GEO cohort, the levels of macrophages, mast cells, neutrophils, para-inflammation, CCR, and type II IFN responses had the similar tendencies with TCGA cohort (Figures 10C,D). Next, the CIBERSORT approach was applied to gain further insight into the composition of immune and stromal cells in the TME of patients with GC in two groups. The results obtained from 375 patients with GC in TCGA and 433 patients in GEO were summarized in Figure 11A. The results indicated that pro-tumor immune cells of M2 macrophages and resting memory CD4 + T cells were found to be higher in the high-risk group (Figures 11B–E). In contrast, the levels of anti-tumor immune cells, such as CD8 + T cells and activated memory CD4 + T cells were increased in the low-risk group (Figures 11F–I). These results provided some clues about the failure of immunotherapy in patients with GC, which need to be verified in subsequent clinical studies with large samples.

## Discussion

Inflammatory etiology attributed to various cancers, such as GC (30). HP infection is regarded as the main contributor to GC and it could cause chronic inflammation of the stomach and leads to atrophic gastritis, intestinal metaplasia, heterogeneous hyperplasia, and GC (5). Inflammation-related genes are considered to be potential prognostic biomarkers for patients with GC.

In our study, we obtained 29 differentially expressed and prognostic inflammation-related genes. Then, 11 inflammation-related gene signatures were constructed by the LASSO algorithm analysis. Among these 11 inflammation-related genes (*APOA1, CYP19A1, F5, HBB, IGFBP1, MATN3, MTTP, PON1, PVT1, RNASE3*, and *SERPINE1*), *APOA1, HBB, and MTTP* were downregulated, the rest of the genes expressed highly in GC tissues. Moreover, we found that the high-risk group was significantly correlated with shorter OS.

Our findings suggested that these inflammation-related genes may serve as prognostic markers and therapeutic targets for GC, and there were numerous studies supporting our results. Ma et al. showed that *ApoA1* was significantly related with the clinical outcome of patients with GC (31). Chong et al. discovered that human *APOA1* expression levels in mice with larger tumors were significantly lower than those in mice with smaller tumors. Moreover, *APOA1* expression levels in the plasma of mice with high tumor burden was lower than that of mice with low tumor burden, indicating that the decrease *APOA1* expression correlated closely with tumor progression (32). Sadeghi-Amiri et al. identified that *CYP1A1* expression increased significantly in GC tissues compared with their normal tissue cohort (33). Wang et al. showed that *CYP1A1* was a possible new molecular target for GC therapy (34). Yang et al. found that *CYP19A1* was a prognostic biomarker of GC (35). Ma et al. showed that high *CYP1A1* expression may be a favorable factor for patients with GC (36). Zhang et al. found that *CYP1A1* was expressed in a comparatively early stage of gastric carcinogenesis and played its parts in the whole process of sequential carcinogenesis (37). Liu et al. found that *F5* was markedly increased in GC tumor tissue and possibly a promising prognostic biomarker (38). Sato et al. showed that an increased expression of *IGFBP1* was more likely to lead to hematogenous metastasis and exhibited a worse survival, *IGFBP1* might be a possible new predictive factor and candidate for molecular targeted therapy in GC (39). Luo et al. demonstrated that there was an increased expression and release of *IGFBP-1* in HP-infected GC cells, and *IGFBP-1* could inhibit the migration of GC cells, which possibly had a protective effect in HP-induced GC (40). Wu et al. confirmed that *MATN3* was highly overexpressed in patients with GC and *MATN3* might serve as an independently predictable prognostic factor for the poor prognosis in patients with GC. (41). Krzystek-Korpacka et al. displayed that the activity of *PON1* was reduced in gastroesophageal cancer, which was paralleled with the level of inflammation and cancer-associated anemia. The reduction of *PON1* appeared to present with lymph node metastasis, whereas *PON1* failed to serve as an independent indicator for clinical application (42). Ding et al. found that *PVT1* expression was higher in GC tissues compared with adjacent non-cancerous tissues and it was correlated with lymph node metastasis in GC. Moreover, *PVT1* exhibited promising therapeutic targets for the treatment of GC and for enhancing paclitaxel sensitivity (43). Kong et al. revealed that an increased expression of *PVT1* was distinctly related to the depth of tumor invasion and late TNM stage and it could act as an independent predictive factor for OS (44). Zhang et al. discovered that *LncRNA PVT1* was overexpressed in cisplatin resistant GC patient tissues and cisplatin resistant GC cells, and its overexpression facilitated the development of multidrug resistance (45). Yuan et al. suggested that the expression of *PVT1* was markedly increased in both GC tissues and cell lines versus normal controls, and there was a notable association between its upregulation and the depth of invasion, advanced TNM, and lymph node metastasis in GC (46). Xu et al. revealed that *LncRNA PVT1* was notably increased in GC tissues and the high *PVT1* expression was closely associated with the poor prognosis in patients with GC (47). Chen et al. found that *circPVT1* was highly increased in GC tissues and it could competitively bind *miR-125* to promote the GC cell proliferation (48). Zhao et al. suggested that *SERPINE1* was considered associated with carcinogenicity and adverse prognosis in GC (49). Yang et al. revealed that GC patients with the high expression of *SERPINE1* were associated with poorer OS and DFS (50). Yang et al. identified that GC patients with higher *SERPINE1* expression had shorter OS and it could promote the proliferation, invasion, and migration of GC cells, indicating that *SERPINE1* might serve as a new therapeutic target for GC (51). Liao et al. revealed that *SERPINE1* and *SPARC* were expressed exclusively higher in GC tissue and they were correlated with an unfavorable outcome (52). These results were consistent with our study in general. There are several genes that have been less studied in GC at present, but possibly could be candidates for further study in the future for new insight into the prognosis and treatment of GC. In addition, our study was analyzed based on the mRNA expression level of differentially expressed and prognostic inflammation-related genes, which had some limitations. The expression levels of proteins which could be regulated by various factors, and the mRNA expression pattern is far away from the real protein expression in the clinical sample (53). Therefore, a multi-center clinical study with a larger sample size needs to be implemented to validate the expression of the hub genes at the mRNA and protein levels, respectively.

Moreover, GSEA analyses suggested that the two different groups of patients with GC were primarily involved in extracellular matrix, which is the component of the TME. In recent years, immunotherapy for GC has achieved promising outcomes, but not all patients can benefit from immunotherapy due to tumor heterogeneity (2). Since the TME is closely related to immunotherapy, understanding the condition of immune cells in the TME and exploring promising therapeutic targets for TME remodeling to relieve the immunosuppressive TME would be beneficial in the treatment of GC. The TME is the partial biological environment in which the tumor develops. The TME is largely made up of immune cells, endothelial cells, fibroblasts, extracellular matrix, and the necessary growth factors, adhesion factors, and oxygen for the cell microenvironment (54). In the TME, tumor-associated immune cells can be divided into two main categories: anti-tumor immune cells and pro-tumor immune cells, and they have different roles in the different stages of tumor progression (55). Anti-tumor immune cells mainly contain CD8 + cytotoxic T cells, effector CD4 + T cells, natural killer cells, DCs, M1 macrophages, and N1 neutrophils. In addition, there are large number of tumor-promoting immune cells, such as regulatory T cells, N2-polarized neutrophils, and M2-polarized macrophages. According to the cell types in the TME, some studies have shown that if tumor-inhibiting cells are predominant in the TME, it could play a role in inhibiting tumor formation by killing tumor cells. On the contrary, if tumor-promoting cells occupy a superior position, the TME could play a pro-tumor activity in promoting the proliferation of tumor cells, thus playing a role in promoting tumors (56). Based on the characteristics of the TME, tumors can be classified into “hot tumor” and “cold tumor.” Tumors characterized as highly immunogenic usually described as “hot tumor” with an immunogenic microenvironment, such as inflated T cells, memory T cells, and cytokines (57). Contrary to “hot tumor,” the low-immunogenic tumors with a non-immunogenic microenvironment and the absence of the above-mentioned components are called “cold tumor.” Numerous reports have demonstrated that only “hot tumor” is able to respond well to immunotherapy, whereas “cold tumor” often undergo immune escape. GC is a typical inflammatory-related malignant tumor whose microenvironment contains a large immune cell. The status and function of tumor-infiltrating immune cells regulates the biological behavior of GC. Therefore, understanding the relationship between the TME and inflammation-related genes of GC is of great importance.

Our study demonstrated that the immune cell subpopulations of DCs, macrophages, mast cells, and neutrophils were upregulated in the high-risk groups. They are responsible for promoting tumor progression in the TME. As for the immune-related pathways, para-inflammation and CCR were markedly upregulated in the high-risk group, which could facilitate tumor development. To further explore the precision of the above results, the CIBERSORT approach was applied to gain further insight into the composition of immune and stromal cells in the TME of GC patients. The results demonstrated that the pro-tumor immune cells of M2 macrophages and resting memory CD4 + T cells were found to be higher in the high-risk group, perhaps this is the reason for their poor prognosis. Besides, the levels of CD8 + T cells and activated memory CD4 T + cells which could inhibit the tumor progression were significantly increased in GC with low-risk score and they have a better prognosis. The above results show that patients with GC in the high inflammatory risk group have the characteristics of “cold tumor” and have a poorer prognosis. Therefore, targeting inflammatory response-related genes and remodeling the TME to turn “cold tumor” into “hot tumor” may be a promising solution to improve the efficacy of immunotherapy for patients with GC.

However, there are still some limitations in our study. First, the prognostic model was constructed based on TCGA data, on which the original samples were probably based on single biopsies, further well-designed studies and a multi-center clinical study with a larger sample size need to be implemented to validate our results. Second, we mainly used the preclinical models to reveal that the inflammation-related genes maybe a prognostic biomarker and involve in the immunosuppressive microenvironment in patients with GC. The larger samples need to be implemented to validate them at the mRNA and protein levels, respectively. Third, we analyzed the correlation between risk score and the different immune cell subpopulations in the TME of patients with GC. Nevertheless, more experiments are needed to provide support for our findings. In future studies, the exact roles of inflammation-related genes in the microenvironment of GC worthy of thorough investigation.

## Conclusion

We constructed a prognostic model based on inflammation-related genes associated with prognosis, and evaluated the proportion of immune cell subtypes in the tumor microenvironment. We found that the 11 inflammation-related genes could serve as prognostic markers and patients with GC in the high inflammatory risk group have the characteristics of “cold tumor” and have a poorer prognosis. These results provided novel ideas for improving the therapeutic efficacy of GC patients by overcoming immunosuppressive tumor microenvironment. However, there are still some limitations in our study. Further well-designed studies and a multi-center clinical study with a larger sample size need to be implemented to support for our findings.

## Code Availability

All codes are available from the corresponding author on reasonable request and we could offer the version information as necessary and any restrictions on availability.

## Data Availability Statement

The original contributions presented in the study are included in the article/Supplementary Material, further inquiries can be directed to the corresponding author.

## Author Contributions

YW and CY: conceptualization. YW and XY: writing the original draft. LG, XX, and YW: methodology, software, and revision. YW, XY, LG, XX, and CY: validation. All authors approved the final version of the manuscript.

## Conflict of Interest

The authors declare that the research was conducted in the absence of any commercial or financial relationships that could be construed as a potential conflict of interest.

## Publisher’s Note

All claims expressed in this article are solely those of the authors and do not necessarily represent those of their affiliated organizations, or those of the publisher, the editors and the reviewers. Any product that may be evaluated in this article, or claim that may be made by its manufacturer, is not guaranteed or endorsed by the publisher.

## Footnotes

1 www.ncbi.nlm.nih.gov/gene, accessed on 13 Apr 2021.

2
http://www.proteinatlas.org/

3
http://www.gsea-msigdb.org/gsea/index.jsp
